# Targeting tumor resistance mechanisms

**DOI:** 10.12703/r/10-6

**Published:** 2021-01-26

**Authors:** Louise Gerard, Laurent Duvivier, Jean-Pierre Gillet

**Affiliations:** 1 Laboratory of Molecular Cancer Biology, Molecular Physiology Research Unit (URPhyM), Namur Research Institute for Life Sciences (NARILIS), Faculty of Medicine, University of Namur, Namur, Belgium

**Keywords:** Therapeutic developments, translatability of preclinical studies, organoids, venoms

## Abstract

Cancer develops resistance to treatments through many mechanisms. Single-cell analyses reveal the intratumor heterogeneity and dynamic relationships between cancer cell subpopulations. These analyses also highlight that various mechanisms of resistance may coexist in a given tumor. Studies have unraveled how the microenvironment affects tumor response to treatments and how cancer cells may adapt to these treatments. Though challenging, individualized treatment based on the molecular characterization of the tumor should become the new standard of care. In the meantime, the success rate of clinical trials in oncology remains dramatically low. There is a need to do better and improve the predictability of preclinical models. This requires innovative changes in *ex vivo* models and the culture system currently being used. An innovative ligand design is also urgently needed. The limited arsenal of medicinal chemistry reactions and the biases of scaffold selection favor structurally similar compounds with linear shapes at the expense of disc and spherical shapes, which leave a large chemical shape space untouched. In this regard, venoms have received increasing interest as a wellspring for drug candidates. Overall, the characterization of tumor heterogeneity has contributed to advancing our understanding of the mechanisms that underlie cancer resistance to treatments. Targeting these mechanisms will require setting key milestones to significantly improve the translatability of preclinical studies to the clinic with the hope of increasing the success rate of clinical trials.

## Introduction

Cancer is expected to become the leading cause of death worldwide. The Prospective Urban Rural Epidemiology (PURE) study revealed that in high-income countries and some upper-middle-income countries, deaths from cancer are now more common than those from cardiovascular disease in individuals who are 35 to 70 years old^1^. The reasons are multifactorial but reflect aging, growth of the population, and socioeconomic development^2^.

Advancements in screening, early diagnosis, and treatment have a major impact on the decrease in the overall cancer mortality rate^3^. However, tumor resistance to treatments remains the greatest challenge in improving the outcomes for patients with cancer^4^. The proof of concept that cancer resistance to chemotherapy could develop after multiple drug doses was made in 1942 by a multidisciplinary team of Yale University pharmacologists and physicians^5–7^ who administered intravenous injections of nitrogen mustard to a patient to treat his lymphosarcoma^8^. This clinical trial revealed what is known as acquired drug resistance. Further studies have shown that only some cancers respond to treatment, unravelling intrinsic resistance. Some 35 years later, Ling and colleagues demonstrated the role of a cell surface glycoprotein, designated as permeability-glycoprotein, in the resistance of Chinese hamster ovary cells to colchicine^9,10^. The authors showed that these cells were also resistant to a wide range of structurally and mechanistically unrelated drugs, which is defined as multidrug resistance^9,10^. Ten more years were necessary to clone the *ABCB1* gene encoding this permeability-glycoprotein^11^. This was the first member of a large superfamily of membrane proteins comprising 48 members divided into seven families, termed ATP-binding cassette (ABC) transporters^12^. Since ABCB1, many other ABC transporters have been associated with drug resistance^13^. Unfortunately, the majority of clinical trials failed to support the modulation of these drug efflux transporters as a therapeutic strategy to overcome ABC transporter–mediated resistance^14^. The toxicity of these inhibitors remains a major issue, among others, which are addressed in some articles^13–15^. The characterization of cancer response to chemotherapy has led to the identification of many additional mechanisms of drug resistance driven by a decreased expression of uptake transporters, epigenetic alterations, drug sequestration, and enhanced DNA damage repair^4^.

Targeted therapy and immunotherapy have emerged over the past two decades. Although these treatments have shown great promise in many cancers, their clinical impact remains limited by the development of cancer resistance mechanisms^16,17^. In this mini-review, we address the current therapeutic developments to overcome the mechanisms of cancer drug resistance. We also propose two key milestones to improve the impact of preclinical research on the clinic.

## A shortcut toward the blueprint of tumor resistance mechanisms

**Figure 1.**
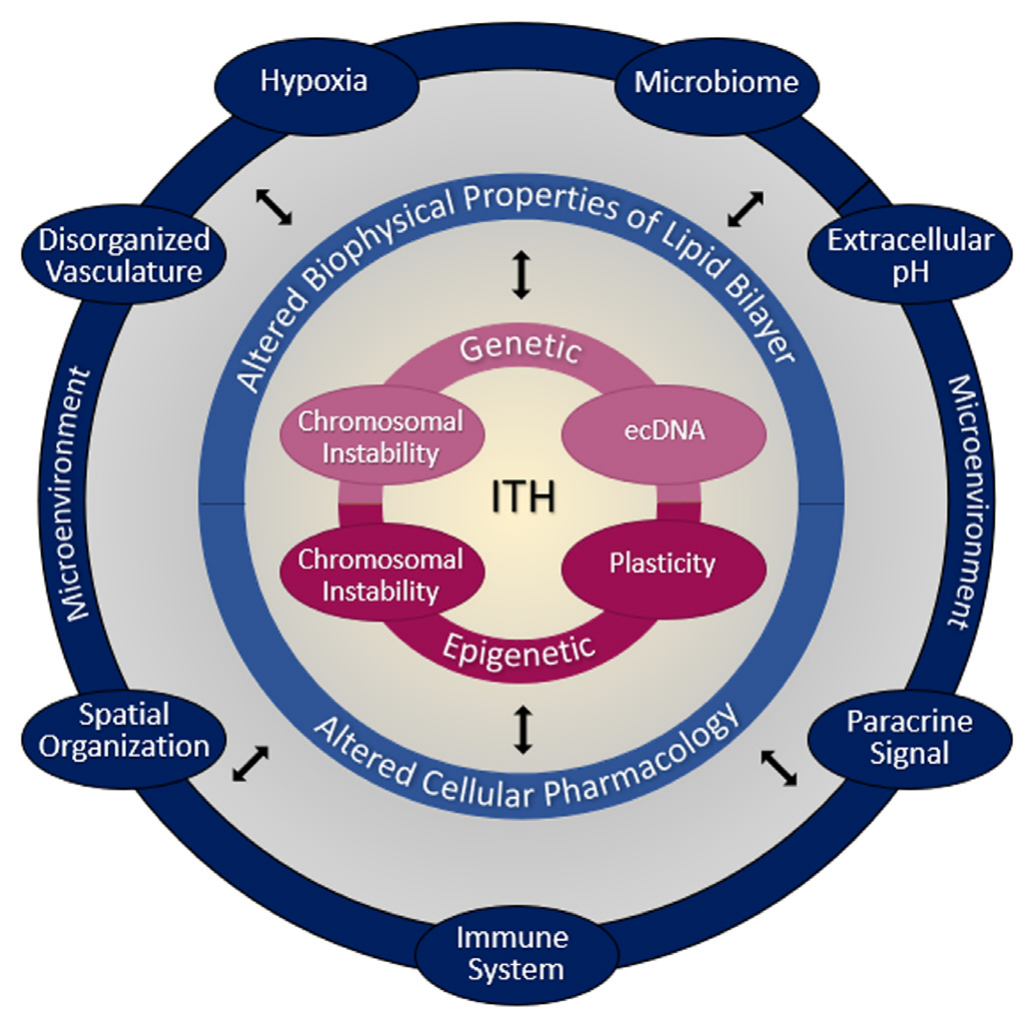
Blueprint of tumor resistance mechanisms. A tumor is composed of genetically and epigenetically heterogeneous cell populations that interact with one another and with the microenvironment. Genetic alterations in signaling pathways that control cell proliferation, apoptosis, DNA repair, or expression of genes mediating treatment resistance are common hallmarks of cancer^29^. These cells often display chromosomal instabilities and carry extrachromosomal DNA (ecDNA). These ecDNAs were shown to drive genetic heterogeneity, promote tumor aggressiveness, and lead to drug resistance^30^. Epigenetic alterations drive cancer phenotype. Mutations have been identified in various classes of epigenetic modifiers involved in DNA methylation, chromatin remodeling, or histone post-translational modifications. Epigenetics also play a key role in the development of resistance mechanisms against anticancer treatments^31^. Perturbations in lipid metabolism lead to alterations in the biophysical properties of the lipid bilayer. Furthermore, cancer cells often express a wide array of drug efflux transporters and uptake transporters, including ATP-binding cassette (ABC) transporters, non-ABC transporters, and solute carriers. Overall, these alterations have a major impact on drug uptake. There is a complex and dynamic interplay between the microenvironment and the tumor through continuous paracrine communication between tumoral and stromal cells. Hypoxia leads to the upregulation of many genes that mediate resistance to treatments, but it may dramatically impact the effectiveness of drugs depending on their redox properties. The acidic extracellular compartment also has important effects on the success of chemotherapy. Likewise, there is a continuous interaction between the tumor and the immune system. Although immunotherapy holds great promise, mechanisms of resistance have been identified, and these inevitably limit the clinical impact of this treatment strategy^16,32^. Lastly, the microbiome has gained much attention over the past years^33^. Studies have revealed that the gut microbiome may influence the outcome of immunotherapy^34,35^ or promote chemoresistance to colorectal cancer^36,37^. Advances in the understanding of intratumor heterogeneity (ITH), the microenvironment, and their complex dynamic interplay will allow us to generate the blueprint of the mechanisms of cancer resistance to treatments.

Tumor development is a Darwinian process that leads to genetically heterogeneous cell populations, which interact with one another and with the microenvironment (Figure 1)^18^. The cancer stem cell (CSC) concept adds another level of complexity. Initially, tumor heterogeneity was speculated to result from its hierarchical organization, sustained by a few quiescent cells, called CSCs, which are resistant to chemo- and radiotherapy. CSCs became the target of choice to cure cancer. Although their elimination remains a challenge, the concept has evolved through extensive studies, which revealed, for instance, the plasticity of both CSCs and non-CSCs^19^. Intra- and intertumor heterogeneity is a key feature to address cancer drug resistance, perhaps the Rosetta stone of therapy resistance, to quote Marusyk and colleagues^20^. Therefore, different techniques have emerged to address tumor heterogeneity. For instance, the characterization of a subpopulation of cancer cells can be achieved by single-cell sequencing^21^. Another next-generation sequencing method, multi-regional exome sequencing, provides insight into tumor mutation evolution^22^. However, multiple biopsy remains a constraint, and combination with a less-invasive approach, such as circulating biomarker analysis (that is, circulating tumor cells, tumor-derived cell-free DNA, and extracellular vesicles), is used to monitor disease development^23^. Following analysis, targeted therapies may be selected on the basis of the mutational profile of the tumor. Altogether, this allows treatment adaptation based on the discovered mutations.

Furthermore, the development of *in vitro* models, such as organoids, remains a promising tool toward a better understanding of the mechanisms leading to tumor heterogeneity^24^. The characterization of cancer heterogeneity will contribute to generating a blueprint of the mechanisms of resistance to treatments.

## Toward therapeutic developments to overcome cancer resistance mechanisms

### Treatment modulation

The use of drugs at their maximal tolerated dose (MTD) has been the gold standard for cancer treatment for years. However, the development of chemotherapy resistance, combined with high off-target toxicity, has led us to rethink the way to use those existing molecules without losing their bioactive effects. This has given rise to the growing interest in the notion of metronomic chemotherapy (MC). MC refers to the constant administration of low doses of a drug without drug-free phases^25^. As mentioned previously, resistance may arise from tumor heterogeneity. Compared with conventional therapies, which target proliferating tumor cells, MC is a multitargeted therapy that mainly targets endothelial cells and tumor vasculature formation, somehow repositioning the initial drug^25^. As endothelial cells are considered genetically stable, they are less susceptible to developing resistance mechanisms^26^. MC also acts against tumor development through the stimulation of the immune system, which is often weakened with MTD-based chemotherapy^26,27^. MC using different classic drugs—such as cyclophosphamide, methotrexate, or docetaxel—has been investigated alone or in combination in clinical trials to fight breast cancer^28^. Unfortunately, major advances in the field have been delayed because of the lack of comprehension of MC mechanisms. However, some have recently been suggested to be more powerful when used as maintenance therapy, as depicted for breast cancer and some pediatric malignancies^38,39^. On the other hand, research on intermittent chemotherapy (sometimes referred to as a treatment holiday) is still ongoing^40,41^. This adapted regimen, including extended drug breaks, would theoretically delay resistance, as suggested by Madan and colleagues for taxane resistance in prostate cancer^42^.

### Counteracting cell plasticity

Drug exposure has been shown to activate different processes drastically changing cell phenotypes, such as transdifferentiation or epithelial-to-mesenchymal transition (EMT), ultimately turning cells into treatment-refractory entities that are likely to include CSCs^43,44^. However, numerous studies with a drug holiday period showed resensitization to the initial treatment, suggesting a reversible phenotype^45,46^. This raised the idea of controlling and reverting the acquired drug resistance to sustain treatment benefits. Indeed, phenotypical changes because of EMT are largely attributed to epigenetic modifications^43^. Three main strategies have been designed to counteract phenotypic changes because of cell plasticity and these have been extensively reviewed^44^. The first consists of preventing tumor cell plasticity. This has been investigated by targeting slow-cycling drug-tolerant cells, thought to be an intermediate step in the cell plasticity process. Drug tolerance of these cells is partially attributed to chromatin modulation. The use of histone deacetylase inhibitors or DNA methylation inhibitors in combination with chemotherapy or immunotherapy may result in an effective treatment for cancer resistance^47,48^. The second strategy aims to target the new cell fate. Here, it relies on taking advantage of the molecular characteristics of the mesenchymal cell. For instance, the tyrosine kinase receptor AXL has been in the spotlight as a *front door* to kill cancer cells undergoing EMT. AXL inhibition leads to cell resensitization to different chemotherapeutics, such as mitotic inhibitors, tyrosine kinase inhibitors, or platinum-based therapies^49^. Finally, the new cell fate is reversed, so promoting mesenchymal-to-epithelial transition (MET) is the third strategy considered. Reactivation of the E-cadherin promoter by cholera toxin and forskolin results in MET induction and resensitization to doxorubicin, paclitaxel, EGFR inhibitors, and proteasome inhibitors^50^. However, this option has to be designed wisely, as it could also promote metastatic colony formation^43^. Differentiation therapy to reverse abnormal stemness signaling pathways in CSCs also constitutes a popular research project to thwart resistance^51^.

### Combinational therapies targeting resistance mechanisms

Combinational therapy requires the characterization of the resistance-driving mechanism. For example, second mitochondria-derived activator of caspases (SMAC) mimetics improve the sensitization of different cancer types to classic chemotherapeutics. SMAC directly binds to X-linked chromosome inhibitors of apoptosis proteins to prevent its inhibitory effect on apoptosis-effector caspases, such as caspases 3, 7, and 9^52,53^. Clinical trials evaluating SMAC mimetics—such as birinapant, LCL161, or debio 1143—as monotherapy revealed a moderate response^54^. However, they showed a significant effect when used as a complement for chemotherapy. A phase II clinical studies combining birinapant and irinotecan has highlighted the potential of the combination to treat irinotecan-refractory metastatic colorectal cancer (NCT01188499)^55^. Another phase II study published promising results on the combination of paclitaxel and LCL161 in triple-negative breast cancer (NCT01617668), supporting the interest in SMAC mimetics in combination with chemotherapy^56^.

### Taking advantage of ABC transporter expression

In the past few years, light energy has become a promising strategy to overcome multidrug resistance. Mao and colleagues developed an antibody targeting ABCB1 conjugated with a photosensitizing agent^57^. Localized light activation, via near-infrared laser irradiation of the photosensitizing agent, leads to tumor-specific cytotoxicity via reactive oxygen species (ROS) production. ROS oxidizes NADH into NAD^+^, which alters the proton gradient across the inner mitochondrial membrane and interferes with ATP synthase. The lack of ATP leads to inactive ABC transporters and cell death. Photodynamic therapy has made its way to clinics in a phase 1/2a trial for the treatment of head and neck cancer (NCT02422979).

### Toward additional potential therapeutic approaches

Mitochondrial transplantation has emerged as a new approach to restore mitochondrial function in a variety of diseases^58^. Patients with myocardial ischemia-reperfusion injury were the first clinical application of mitochondrial transplantation^59^. After treatment, all patients showed improved myocardial systolic function. In cancer cell lines, mitochondrial transplantation has been shown to restore impaired mitochondrial function, which impacts chemoresistance and cancer proliferation^60–62^. The anticancer effects were shown to be independent of the toxicity mediated by the transplantation methods. However, as highlighted by Gollihue and colleagues, injection of the mitochondria into tissue could lead to immune response and inflammatory reaction even if not reported in different animal models^63^. Moreover, incorporation mechanisms are still rarely described^63^. There is a long way to go before the clinical application of mitochondrial transplantation in cancer treatment.

It is now well established that microRNAs (miRNAs) regulate numerous pathways, including oncogenic-driving processes. This highlights their potential as targets, using anti-miRs or miRNA mimics, known as miRNA replacement therapy. Anti-miRs specifically bind and inhibit miRNA, while miRNA mimics restore the function of a silenced miRNA^64^. In a phase I clinical trial (NCT01829971), the safety of miR-34 mimics, replenishing the function of the endogenous miR-34 family involved in p53-related DNA damage response and apoptosis, is being evaluated in patients with solid tumors and hematologic malignancies^64,65^. However, this therapeutic strategy may lead to toxicity through off-target or on-target activity in other tissues and immunogenic reaction to the RNA itself or to the excipient used in the delivery system. Therefore, using miRNAs as therapeutics requires further optimization and extensive monitoring^66^.

The concept of synthetic lethality consists of a synthetic lethal pair, in which one is a gene product with a cancer-specific mutation and the other one is the drug target^67^. A clinical example of this concept is the use of PARP inhibitors to treat *BRCA*-mutant ovarian cancers^68^. These inhibitors have limited toxicity on normal cells carrying at least one copy of the *BRCA* gene. This is of particular interest, as it currently constitutes the only way to take advantage of tumor suppressor gene loss. The development of CRISPR technologies has helped set up in-depth screening to discover new synthetic lethal pairs^67^.

We can also cite the development of proteolysis-targeting chimeric molecules (PROTACs). PROTACs are made up of a ligand of the target protein and of an E3 ligase recruiting element, attached together by a linker^69,70^. The system degrades the protein of interest by using the endogenous ubiquitin proteasome system^69,70^. Two PROTACS against the androgen receptor and the estrogen receptor (called ARV-110 and ARV-471, respectively) recently reached phase I clinical trial (NCT03888612 and NCT04072952), underlining the interest in this new therapeutic strategy.

## Setting milestones

Time has come for a new paradigm shift in the way we think of preclinical models. The last one occurred more than 30 years ago with the development of the NCI-60 panel of cancer cell lines^71,72^. The major objective of this change was to improve the limited predictability of transplantable murine neoplasms for solid tumors. Since then, larger cancer cell line panels have been created to reflect the genomic diversity of human cancers^73,74^. In a recent study, the probability of success of a clinical trial in oncology was estimated to be 3.4%^75,76^. Many reasons may explain why most clinical trials fail^77^. Among these, the predictability of preclinical models remains limited. At this stage, we should shift from incremental improvements of these models, which have been observed over the past 30 years, to an innovative change. *Ex vivo* models that recapitulate the intra- and intertumor heterogeneity in a culture system that better simulates the *in vivo* situation are needed^78,79^. In this regard, human pluripotent stem cell– or adult stem cell–derived organoid models showed great potential in modeling human diseases^80^. Cancers have been studied using organoids generated either from the genetic engineering of stem cells or directly from tumor biopsy^81,82^. Efforts have been directed toward mimicking the tumor microenvironment and developing organoids-on-a-chip^83,84^. In a recent study, Koike and colleagues demonstrated the feasibility of using human organoids to study the communication between different organs, more precisely the liver, the pancreas, and the gastrointestinal tract^85^. It was also shown that the vascular network can be generated in these models^86^. Technology has evolved, and sophisticated chamber devices have been proposed^87–89^. It is not an exaggeration to say that there is currently no unsurmountable obstacle for recapitulating the complexity of cancers and integrating these powerful models into preclinical research.

A limited number of reactions also dominate the chemical landscape of medicinal chemistry, which, combined with the biases of scaffold selection, favor structurally similar compounds with linear shapes. There is a compelling need for an innovative ligand design^90^. Perhaps one way out would consist of putting more effort toward the characterization of venoms^91,92^. It is estimated that 200,000 venomous species exist, which represent around 40 million toxins; of these, fewer than 5,000 have been pharmacologically characterized. Venomics—which integrate genomic, transcriptomic, and proteomic approaches to the study of venoms—is gaining interest as a strategy for drug discovery^93–95^. Tozuleristide uses chlorotoxin, a 36–amino acid peptide isolated from the venom of the deathstalker scorpion *Leiurus quinquestriatus*, and an infrared dye^92^. It is being evaluated as a new diagnostic drug in a phase II/III clinical trial to determine how well the drug can help distinguish between tumor and normal tissue during surgery in pediatric primary central nervous system tumors (NCT03579602). What is unique with this peptide is that it is retained in the tumor and can cross the blood–brain barrier^96^. Chlorotoxin molecular targets include the CLC-3 voltage-gated chloride channel, annexin-2, and matrix metalloproteinase-2^96–99^. SOR-C13 is a carboxy-terminal truncation of soricidin, a 54-residue paralytic peptide found in the venom of the short-tailed shrew *Blarina brevicauda*. This peptide blocks Ca^2+^ uptake via inhibition of TRPV6 channel^100^. A phase I trial revealed that SOR-C13 was safe and generally well tolerated in patients with advanced tumors of epithelial origin (NCT01578564). The study also suggested that SOR-C13 has anticancer activity with stable disease observed in more than half of the patients evaluated^101^. Many other peptides have been shown to have an anticancer effect, but the majority of these studies are still at the very preliminary stage^102–105^. Burkholderia lethal factor 1 (BLF1) is a monomeric toxin from the bacteria *Burkholderia pseudomallei*, which inhibits translation initiation by inactivation of eukaryotic initiation translation factor 4A (eIF4A) through deamidation of the glutamine 339^106^. BFL1 was shown to selectively induce apoptosis in MYCN-amplified neuroblastoma cell lines^107^. It is worth mentioning that Tv1, a venom peptide from the marine snail *Terebra variegata*, was shown to selectively kill hepatocellular carcinoma cells in syngeneic tumor-bearing mice. The proposed mechanism of action includes the binding of Tv1 to TRPC6 and/or the TRPV6 channel, which leads to calcium-dependent apoptosis^108^. To the best of our knowledge, neither BFL1 nor Tv1 has entered clinical trials. This library of natural products, mostly unexplored, holds great promise for the treatment of cancer, especially for those who are intrinsically resistant to chemotherapy or develop resistance to targeted therapy, such as the case with hepatocellular carcinoma^109^.

These are two key milestones that can realistically be achieved in the very near future. From there on, we may reasonably speculate that the number of compounds reaching clinical practice should increase tremendously.
